# Yale Global Tic Severity Scale (YGTSS): Psychometric Quality of the Gold Standard for Tic Assessment Based on the Large-Scale EMTICS Study

**DOI:** 10.3389/fpsyt.2021.626459

**Published:** 2021-02-25

**Authors:** Martina Haas, Ewgeni Jakubovski, Carolin Fremer, Andrea Dietrich, Pieter J. Hoekstra, Burkard Jäger, Kirsten R. Müller-Vahl, The EMTICS Collaborative Group, Alan Apter

**Affiliations:** ^1^Clinic of Psychiatry, Social Psychiatry, and Psychotherapy, Hannover Medical School, Hannover, Germany; ^2^Department of Child and Adolescent Psychiatry, University Medical Center Groningen, University of Groningen, Groningen, Netherlands; ^3^Clinic of Psychosomatics and Psychotherapy, Hannover Medical School, Hannover, Germany

**Keywords:** Tourette's syndrome (TS), YGTSS = Yale Global Tic Severity Scale, psychometric properties, internal consistency, confirmatory factor analysis

## Abstract

The Yale Global Tic Severity Scale (YGTSS) is a clinician-rated instrument considered as the gold standard for assessing tics in patients with Tourette's Syndrome and other tic disorders. Previous psychometric investigations of the YGTSS exhibit different limitations such as small sample sizes and insufficient methods. To overcome these shortcomings, we used a subsample of the large-scale “European Multicentre Tics in Children Study” (EMTICS) including 706 children and adolescents with a chronic tic disorder and investigated convergent, discriminant and factorial validity, as well as internal consistency of the YGTSS. Our results confirm acceptable convergent and good to very good discriminant validity, respectively, indicated by a sufficiently high correlation of the YGTSS total tic score with the Clinical Global Impression Scale for tics (*r*_*s*_ = 0.65) and only low to medium correlations with clinical severity ratings of attention deficit/hyperactivity symptoms (*r*_*s*_ = 0.24), obsessive–compulsive symptoms (*r*_*s*_ = 27) as well as internalizing symptoms (*r*_*s*_ = 0.27). Internal consistency was found to be acceptable (Ω = 0.58 for YGTSS total tic score). A confirmatory factor analysis supports the concept of the two factors “motor tics” and “phonic tics,” but still demonstrated just a marginal model fit (root mean square error of approximation = 0.09 [0.08; 0.10], comparative fit index = 0.90, and Tucker Lewis index = 0.87). A subsequent analysis of local misspecifications revealed correlated measurement errors, suggesting opportunities for improvement regarding the item wording. In conclusion, our results indicate acceptable psychometric quality of the YGTSS. However, taking the wide use and importance of the YGTSS into account, our results suggest the need for further investigations and improvements of the YGTSS. In addition, our results show limitations of the global severity score as a sum score indicating that the separate use of the total tic score and the impairment rating is more beneficial.

## Introduction

Tics are defined as rapid, repetitive, non-rhythmic movements or vocalizations (1). The nature of tics with their heterogeneous presentation as well as waxing and waning course is complex (2) and an accurate assessment of tic severity is thus challenging (3). The Yale Global Tic Severity Scale [YGTSS (4)] is a semi-structured clinical interview and currently the gold standard for assessing the severity of tics in children and adults (5). The YGTSS enables evaluations of number, frequency, intensity, complexity, and interference of motor and phonic tics, covering the past week. Each domain is scored on a 6-point scale (range 0–5) with a separate rating for “overall impairment” regarding the subject's daily life and activities (4). Five sum scores can be created: the total motor tic score (range 0–25), the total phonic tic score (range 0–25), the total tic score (TTS; sum of the total motor tic score plus the total phonic tic score), the overall impairment rating (one item; range 0–50), and the global severity score (GSS; sum of the TTS plus the overall impairment rating, range 0–100). Recommendations which score is preferable as a primary endpoint in studies or for the assessment of individual cases in clinical practice have not yet been formulated. However, most studies use the TTS for the assessment of tic severity.

Since an assessment instrument provides the basis for all subsequent evaluations and results, it should prove to be both reliable and valid, as verified by psychometric investigations (e.g., examination of internal consistency, factorial as well as convergent and discriminant validity).

Previous studies have demonstrated very good internal consistency of the YGTSS (4, 6–8). However, previous studies were limited by the use of Cronbach's Alpha (α) for the calculation of internal consistency, since it has numerous well-documented deficiencies (9–12). Most importantly, a violation of the assumptions underlying the use of α (e.g., uncorrelated errors) may lead to strongly biased results (13–18). Accordingly, Lee J. Cronbach himself criticized the extensive use of α (19), particularly since there are alternative measures available recommended by experts. If the sample and the number of items are sufficiently large, McDonald's Omega (Ω) is recommended (20–22), as it does not require the fulfillment of assumptions, such as uncorrelated errors, and is therefore not as susceptible to producing biased results.

Factorial validity is examined by factor analyses, which can be divided into exploratory factor analyses (EFA) and confirmatory factor analyses (CFA). EFA's are mainly used to reduce data and identify a previously unknown factor structure. For example, an EFA is useful in questionnaire development to reduce the initial item pool and to examine the structure and number of underlying latent factors. CFA's aim to test a theoretically assumed factor structure. Thus, if there is already a theory about the factor structure (which may have arisen from a previous EFA), a CFA can confirm (or disprove) this assumed factor structure (23). Only two studies have examined the factor structure of the YGTSS. Using principle component analysis (in a broader sense a form of EFA), Leckman et al. (4) found a two-factor structure (factor 1: motor tics; factor 2: phonic tics) of the YGTSS. However, the factors of the model accounted for only 8% of the total item variance, which leaves most part of the variance unexplained. Only one study attempted to confirm the proposed factor structure using CFA (7). In line with the authors' expectations, items intended to measure motor tics showed high loadings on the “motor factor” and items intended to measure phonic tics demonstrated high loadings on the “phonic factor.” In addition, inter-factor correlations were as expected. However, the model showed only a poor to modest fit.

Considering the results on internal consistency (measured with α) on the one hand and factorial validity on the other hand, the following discrepancy becomes evident: Due to the high loadings (4, 7) of the items in the factor model and the high internal consistency (4, 6–8), a good to very good model fit would be expected. However, contrary to this, the model fit was shown to be only poor to modest (7). The authors suggested the small sample size of their study (*n* = 76) as a possible reason for this discrepancy, which may have had a negative impact on the model fit (7). They recommended replication in a larger sample, yet, to date, this is lacking.

Other aspects of validity are convergent and discriminant validity. Convergent validity, shown by a high correlation of the instrument with measures that are supposed to measure the same construct (in this case tic severity) was usually examined by correlating the YGTSS scores with the Clinical Global Impression Scale Severity (CGI-S) for tics (24). Discriminant validity, shown by low or moderate correlation of the instrument with measure that are supposed to measure other constructs, was often examined by correlating the YGTSS scores with severity assessments of comorbid symptoms, e.g., attention deficit/hyperactivity disorder (ADHD) symptoms (25) or obsessive–compulsive disorder (OCD) symptoms (26) as well as severity assessments of common co-occurring behavioral problems like internalizing symptoms (27). Both convergent and discriminant validity of the YGTSS have been investigated extensively with excellent results in small samples (*N* = 28–105) (4, 6, 7, 26).

In 2018, McGuire et al. (8) suggested some minor revisions of the YGTSS (YGTSS-R) to selected anchor point descriptions (especially regarding “frequency” and “complexity”) to promote using the full range of scores in the domains. This was based on the distribution of the YGTSS items in a large age-mixed sample (516 children and 101 adults). However, aspects of validity were not examined in this study.

In summary although several psychometric properties of the YGTSS have been examined previously, most of these studies included only small samples or used limited analyses. In addition, factorial validity has hardly been investigated so far. A thorough investigation of the psychometric properties is important, because of the widespread use of the instrument and its status as the gold standard for tic assessment (5). The present study aimed to overcome the limitations of previous studies and to supply more reliable information on the psychometric properties of the YGTSS. Therefore, we utilized a subsample of the large-scale “European Multicentre Tics in Children Study” (EMTICS) (28) including 706 children and adolescents as well as adequate statistical analyses to investigate internal consistency, factorial validity, as well as convergent and discriminant validity of the YGTSS.

## Materials and Methods

### Participants and Measurements

We used the large dataset of EMTICS, which is a longitudinal study that has been conducted between 2013 and 2018 in 16 European clinical centers and primarily aimed to examine the role of genes, autoimmunity, and psychosocial stress on the onset and course of tics. In EMTICS, children and adolescents with Tourette's syndrome (TS) or chronic motor or vocal tic disorder (“course cohort”) and their siblings without any tic disorder (“onset cohort”) were examined at several points in time. In the present study, we used all participants of the “course cohort” in the baseline sample with an established diagnosis of TS or chronic motor or vocal tic disorder according to DSM IV-TR criteria (29), resulting in a sample of 706 children and adolescents between 3 and 16 years. Rater training was conducted for all clinical assessments with a focus on the YGTSS. Furthermore, throughout the duration of the study regular meetings with all collaborators took place to standardize the procedures (28). For a detailed description of the study design and procedure we refer to Schrag et al. (28).

In the present study we used the data of the YGTSS as well as of the following assessments:

- The Clinical Global Impression Scale Severity [CGI-S (24)] for tics: A clinician-administered single-item tic assessment over the past week (“How severe have the tics been during the week preceding this visit?”), rated on a 7-point Likert scale (range 1–7).
- The Children's Yale–Brown Obsessive Compulsive Scale [CY-BOCS (30)]: A semi-structured clinician-administered interview assessing the severity of obsessions and compulsions occurring over the past week across five areas (time, interference, distressing nature, effort to resist, control over obsessions and compulsions). We used the total OCD severity score (range 0–40) including all 10 items rated on a 5-point Likert scale (range 0–4).
- The Swanson, Nolan, and Pelham, version IV scale [SNAP-IV (31)]: A questionnaire rated by parents assessing the severity of ADHD symptoms over the past week, consisting in total of 26 items on a 4-point Likert-type scale (range 0–3). We used the composite score for ADHD symptoms (18 items; 9 items for inattention, 9 items for hyperactivity/impulsiveness) without the 8 items assessing symptoms of Oppositional Defiant Disorder.
- The Strengths and Difficulties Questionnaire [SDQ (32)]: A questionnaire rated by parents assessing strengths and difficulties over the past 2 weeks across five areas (emotional problems, conduct problems, hyperactivity, peer problems, prosocial behavior), consisting in total of 25 items on a 3-point Likert-type scale (range 0–2). We used the severity score for internalizing symptoms (10 items; 5 items of the emotional problems subscale, 5 items of the peer problems subscale).

### Statistical Analyses

All data analyses were carried out using R version 3.4.4.

To investigate a possible influence of age, analyses were performed in the total sample (*n* = 706) as well as in a subgroup of children and adolescents ≥8 years (*n* = 578). To check for differences between the centers, we conducted a pair-wise comparison (Scheffe's test) of the average TTS of the 16 centers (following an ANOVA). In addition, all analyses were performed using the TS subsample (*n* = 624) to check for a possible bias by combining all types of chronic tic disorders.

We calculated descriptive data of all YGTSS items and subscales including mean, standard deviation, median, range and skewness. The ideal would be a skewness of 0. Positive values indicate a right skewed distribution (i.e., greater use of lower scale scores), negative values indicate a left skewed distribution (i.e., greater use of higher scale scores).

To investigate the proposed factor structure (4) we used a CFA and formulated a two-factor model: The five items comprising each of the motor and phonic tic subscales were loaded onto motor and phonic tic factors, respectively. The overall impairment rating was allowed to freely load on both. We ran the CFA using the diagonally weighted least-squares estimator (WLSMV), an estimator suited for analyzing ordinal data.

As fit indices, we used the root mean square error of approximation (RMSEA), the comparative fit index (CFI), and the Tucker Lewis index (TLI). So far, no generally accepted cut-off values for sufficient fit have been defined. For the RMSEA, recommended cut-off values vary between RMSEA ≤ 0.06 (33) and RMSEA ≤ 0.10 (34), for the CFI, between CFI ≥ 0.90 (35) and CFI ≥ 0.95 (33), and for the TLI, between TLI ≥ 0.90 (36) and TLI ≥ 0.95 (33). These indices reflect the global fit of a model. To investigate possible local misspecifications, we additionally used the framework proposed by Saris et al. (37). Significant local misspecifications indicate where deviations from the à priori specified relationships between items and latent factors occur.

Internal consistency was assessed by calculating α and Ω (20). For both, α and Ω the acceptable limit for multidimensional scales would be 0.50 (38).

Convergent validity was determined by Spearman's rank correlations (*r*_*s*_) between YGTSS sum scores and the CGI-S. Discriminant validity was determined by Spearman's rank correlations (*r*_*s*_) between YGTSS sum scores and the total OCD severity score of the CY-BOCS, the composite score for ADHD symptoms of the SNAP-IV as well as the severity score for internalizing symptoms of the SDQ, respectively. Since the YGTSS should not assess OCD, ADHD or internalizing symptoms, but they often co-occur, low to moderate correlations were expected.

## Results

After conservatively cleaning the data and excluding all subjects from the EMTICS “course cohort” baseline sample which did not meet the criteria for a diagnosis of TS or chronic motor or vocal tic disorder, the sample consisted of 706 children and adolescents (541 boys, 165 girls) with a mean age of 10.67 years (*SD* = 2.81 years, range 3–16 years). For none of the analyses an age dependency was detected when comparing the results of the subgroup (>8 years) with the results of the total sample. The analysis of differences between centers showed no distinct site differences regarding the TTS (data not shown). Furthermore, there were no substantial differences between the subsample of TS patients and the total sample (data not shown).

Descriptive data of all YGTSS items and subscales are displayed in Table 1. The means of the TTS items ranged from 0.91 (phonic: complexity) to 3.40 (motor: frequency). All five phonic tic items were below the theoretical scale midpoint of 2.5 with the items 4 (complexity) and 5 (interference) in particular showing a right-skewed distribution with values >1 (but not exceeding 2). Concerning the motor tic items, two items were below and three above the theoretical scale midpoint of 2.5. The values for skewness were all <1. The mean of the overall impairment item was 14.58 and clearly below the theoretical scale midpoint of 25.

**Table 1:** Descriptive data of all YGTSS items as well as YGTSS subscales.

		**Mean**	***SD***	**Median**	**Range**	**Skew**
Motor	Number	2.68	1.18	2	0–5	0.00
	Frequency	3.40	1.25	4	0–5	−0.74
	Intensity	2.75	1.03	3	0–5	−0.52
	Complexity	1.89	1.35	2	0–5	−0.05
	Interference	1.61	1.20	1	0–5	0.63
Phonic	Number	1.35	1.10	1	0–5	0.67
	Frequency	2.16	1.62	2	0–5	−0.01
	Intensity	1.80	1.37	2	0–5	0.12
	Complexity	0.91	1.37	0	0–5	1.24
	Interference	1.06	1.17	1	0–5	1.12
Total motor tic score		12.32	4.60	13	0–23	−0.37
Total phonic tic score		7.27	5.56	7	0–22	0.25
Total tic score (TTS)		19.60	8.62	19	0–44	0.15
Overall impairment rating		14.58	11.94	10	0–50	0.59
Global severity score (GSS)		34.17	18.29	33	0–92	0.42

Overall, 624 subjects met diagnostic criteria for TS, 76 for chronic motor tic disorder, and six for chronic vocal tic disorder. However, not all subjects diagnosed with TS had both motor and phonic tics during the previous week. A total of 178/706 participants were found to have no phonic tics and 21/706 no motor tics within the previous week.

### Internal Consistency

For the internal consistency, different results were found depending on the measure considered: When Ω was considered, the TTS had a value of Ω = 0.58 and the GSS had a value of Ω = 0.56, thus exceeding the acceptable limit of 0.50 for multidimensional scales (38) and indicating sufficient internal consistency. Using α, higher values were found (α =0.87 for TTS, α =0.88 for GSS) indicating a high internal consistency (39).

### Factor Structure

**Figure 1:**
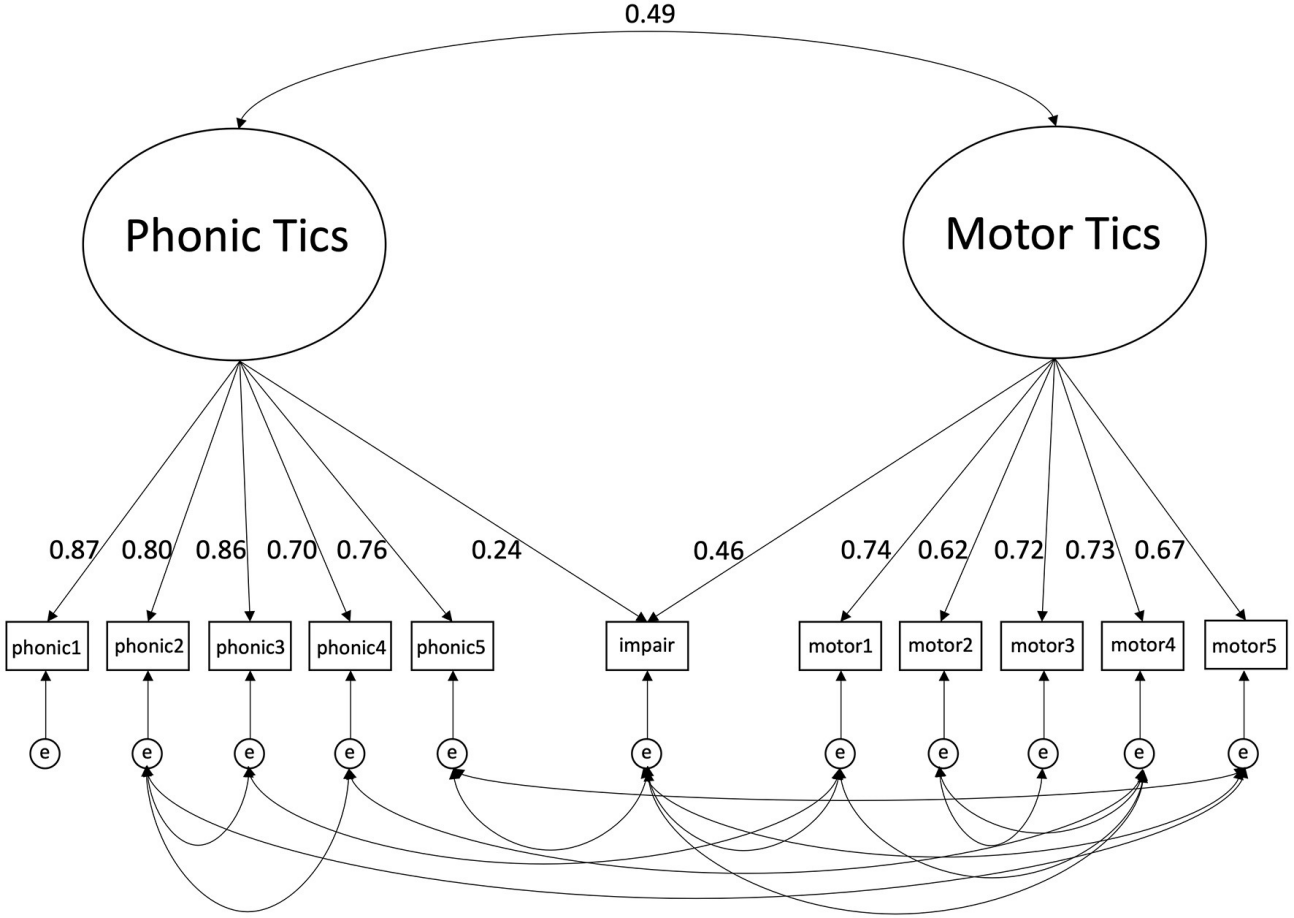
Two-factorial structure of the YGTSS including loadings, inter-factor correlation and correlated errors. phonic1, phonic tics: number; phonic2, phonic tics: frequency; phonic3, phonic tics: intensity; phonic4, phonic tics: complexity; phonic tics: interference; motor1, motor tics: number; motor2, motor tics: frequency; motor3, motor tics: intensity; motor4, motor tics complexity; motor5, motor tics: interference; impair, overall impairment rating; e, error. Results of the Confirmatory Factor Analysis are shown including loadings of the phonic and motor items on the specified factors. In addition, loadings of the overall impairment rating are illustrated, which was allowed to load on both factors. Finally, inter-factor correlation and significant correlations between measurement errors of the items are displayed.

Using the estimator for ordinal data (WLSMV), the following fit indices were obtained for the proposed two factor-model model: RMSEA = 0.09 [0.08; 0.10], CFI = 0.90, TLI = 0.87 indicating a marginal fit. As displayed in Figure 1, motor tic items showed medium to high loadings between 0.62 and 0.74 on the “motor tics factor,” and phonic items showed high loadings between 0.70 and 0.87 on the “phonic tics factor.” The overall impairment rating showed a low to medium loading of 0.46 on the “motor tics factor” and a very low loading of 0.24 on the “phonic tics factor.” The correlation between the two factors was 0.49.

In the subsequent evaluation of local misspecifications, significantly correlated errors between the items (between and within factors) were found. The error correlations of at least 13 item pairs were clearly identified as misspecifications (see Supplementary Material for detailed information).

Due to the only marginal fit, we subsequently conducted an EFA, but also found a two-factor solution and failed to identify a satisfying interpretable alternative model (data not shown).

### Convergent and Discriminant Validity

As shown in Table 2, correlations of the YGTSS sum scores with the GCI-S for tics range from *r*_*s*_ = 0.50 to *r*_*s*_ = 0.65, indicating a sufficient convergent validity (≥0.50), but not reaching an excellent convergent validity of >0.70 (40). As expected, correlations with the total OCD score of the CY-BOCS, the composite score for ADHD symptoms of the SNAP-IV and the severity score for internalizing symptoms of the SDQ were low to moderate (*r*_*s*_ = 0.21–0.27 for the total OCD severity score; *r*_*s*_ = 0.20–0.25 for the composite score for ADHD symptoms; *r*_*s*_ = 0.17–0.30 for the severity score for internalizing symptoms), suggesting adequate discriminant validity (see Table 2).

**Table 2:** Spearman rank correlations (*r*_*s*_) between the YGTSS sum scores and the CGI-S as well as clinical measures of severity of ADHD symptoms and OCD symptoms.

	**Total motor tic score**	**Total phonic tic score**	**TTS**	**Overall impairment**	**GSS**
CGI-S	0.62^**^	0.50^**^	0.65^**^	0.52^**^	0.64^**^
CY-BOCS	0.22^**^	0.25^**^	0.27^**^	0.21^**^	0.26^**^
SNAP-IV	0.20^**^	0.22^**^	0.24^**^	0.21^**^	0.25^**^
INT	0.17^**^	0.28^**^	0.27^**^	0.26^**^	0.30^**^

*TTS, Total Tic Score; GSS, Global Severity Score; CGI-S, Clinical Global Impression Severity; CY-BOCS, Children's Yale–Brown Obsessive–Compulsive Scale; SNAP-IV, Swanson, Nolan, and Pelham IV scale; INT, Internalizing Symptoms of the Strengths and Difficulties Questionnaire (SDQ)*;

** *p < 0.001*.

## Discussion

This study aimed to investigate the psychometric quality of the YGTSS in a large sample to overcome limitations of previous studies. Our results confirm a factor structure with the two factors “motor tics” and “phonic tics” with five items each. Loadings of the impairment rating item were low, supporting the separate use of the TSS and the impairment rating instead of the sum score GSS. However, correlated measurement errors were identified which worsened the model fit and offered possible explanations for inconsistent results concerning factorial validity and internal consistency arising from previous studies. Internal consistency was found to be lower than in previous studies, but still acceptable. Convergent validity was acceptable and discriminant validity was good to very good indicating that the YGTSS is suitable to differentiate between tics and comorbid conditions such as ADHD and OCD as well as frequently co-occurring problems like internalizing symptoms.

Our analysis of the factor structure confirms that the five “motor items” as well as the five “phonic items” each represent separate common factors. However, the loadings of the overall impairment rating (that was allowed to load on both factors) were low, suggesting that this item does not sufficiently “fit” the others, i.e., they probably do not measure the same construct. When combining all items by calculating the GSS, the overall impairment rating has a considerable influence, since its intervals between anchor points differ from the other items (0–50 instead of 0–5) leading to a contribution of 50% to the GSS. Especially in view of this fact, our findings support the current clinical practice not to merge the impairment rating item with the TTS.

Altogether, the model reached a marginal fit that was just above the threshold for acceptable fit. Despite the low loadings of the impairment rating, a much better fit would have been expected due to the high loadings of all other items on the respective factors. Similar to Storch et al. (7), we demonstrated the unusual combination of a marginal fit despite mostly high loadings. While Storch et al. (7) assumed that this was due to their small sample, for the present study this explanation can be excluded since we included a large sample. In order to go a step further, we therefore investigated possible local misspecifications and identified significant correlated measurement errors that can be regarded as a plausible reason for the worsened fit of the model.

Moreover, the finding of correlated measurement errors also offers a possible explanation for the results regarding the analysis of internal consistency: An important assumption for using α is that the errors of the items are uncorrelated, as α can be strongly overestimated by many positively correlated errors (12, 41, 42). The use of Ω does not require uncorrelated errors, so there is no reason to assume that the results for Ω are biased by correlated errors. Accordingly, we found a clear discrepancy between α and Ω probably demonstrating the bias when using α under unfulfilled assumptions. Using Ω, the internal consistency turned out to be markedly lower than for α, but still acceptable.

Considering the results for factorial validity and internal consistency, our data suggest that discrepant previous findings demonstrating on the one hand high internal consistency (4, 6–8) and high loadings of items on the respective factors (7), but on the other hand insufficient model fit (7), could be related to correlated measurement errors. Based on our results, it can be hypothesized that in previous studies not only the internal consistency has been overestimated by using α under unfulfilled assumptions, but also correlated measurement errors worsened the model fit.

Consequently, it is important to understand potential causes of these correlated measurement errors and the possible implications they could have for the YGTSS. In general, the presence of several correlated measurement errors indicates that the affected items share systematic variance independent of the specified tic severity due to the influence of one or even more external factors. This may lead to limitations in the accuracy of the assessment instrument and may therefore have a serious confounding influence on empirical results (43). However, systematic error variance may be acceptable and error correlations can be included in the model (23) if there is an “innocuous” explanation (44). For example, when using different assessment modalities (i.e., self-report, observation) “method covariance” can occur, which can be added to the model as an additional method factor. The integration of correlated measurement errors is critical if no theoretical justification can be found and they are simply added *post hoc* without testing the revised model on a new dataset (45, 46). Since we identified a large number of correlated errors of the YGTSS without any clear pattern, no theoretical justification was possible. When speculating about such correlated errors, it is plausible that the item “number” of tics (item 1) is probably a precondition for the item “complexity” of tics (item 4), which would represent a shared variance that is unrelated to overall tic severity. In addition, it can be assumed that correlated errors are (at least partly) caused by additional problematic influences. Interviewers may have difficulties differentiating between the different domains of the YGTSS. This could particularly affect less experienced raters since the YGTSS was designed for use by highly trained and experienced interviewers (47).

However, in general, the higher the complexity of a scale, the more individuals tend to respond consistently to similar questions that are actually intended to assess different aspects (44) resulting in a consistency effect. Accordingly, the finding of correlated errors should always give reason to check whether the complexity of a scale can be reduced. This is in line with general considerations in scale development including the recommendation to avoid any confusion and complexity wherever possible (48, 49). Accordingly, rating scale categorizations (e.g., anchor points) should be well-defined, mutually exclusive, univocal and exhaustive (50). The anchor point descriptions of the YGTSS do not entirely meet these criteria. For example, for the item “frequency” of tics (item 2), the definitions of category 2 “occasionally” (“Specific tic behaviors are usually present on a daily basis, but there are long tic-free intervals during the day. Bouts of tics may occur on occasion and are not sustained for more than a few minutes at a time”) and category 3 “frequent” (“Specific tic behaviors are present on a daily basis. Tic free intervals as long as 3 h are not uncommon. Bouts of tics occur regularly but may be limited to a single setting”) have the following limitations: They are relatively long, they are largely overlapping and therefore difficult to distinguish, and they use imprecise wordings such as “not uncommon.” In addition, multiple different aspects are combined in one single item such as presence on a daily basis, tic-free intervals, and limitation to settings.

The analysis of the discriminant and convergent validity showed good to very good or acceptable results. The results of the discriminant validity of the YGTSS sum scores were in line with our expectations demonstrating low to medium correlations with assessments of ADHD, OCD, and internalizing symptoms. The correlations of the YGTSS sum scores with the CGI-S for tics (as measurement of convergent validity) suggest sufficient convergent validity (≥0.50), but do not reach an excellent convergent validity of >0.70 (40). However, it must be taken into account that—by definition—it is difficult to determine convergent validity for an assessment considered the gold standard measurement such as the YGTSS. In addition, the CGI-S was the only rating used to examine the convergent validity. Therefore, it would be beneficial to use further measures of convergent validity like self-assessments or parent-rated instruments (e.g., Tourette's Disorder Scale—Parent Rated [TODS–PR (51)] as well as to pay more attention to other forms of validity—such as predictive validity—in the future.

The recently published YGTSS-R included minor modifications primarily concerning the domains “frequency” and “complexity” of tics (8). These changes have been suggested because for both domains skewed distributions in the same direction were found not only for motor, but also for phonic tic items: For the domain “frequency,” both motor and vocal tics showed a negative skewness (greater use of higher scale scores) and for the domain “complexity,” both motor and vocal tics showed a positive skewness (greater use of lower scale scores) (8). Since we found similar (although less pronounced) results for the items of “complexity,” the proposed modifications seem to be reasonable. However, regarding the domain “frequency,” our results showed a skewed distribution for the motor, but not the phonic item, which in turn is an argument against modifying these anchor point descriptions. When taking all items of the YGTSS into consideration, similar to McGuire et al.'s data (8) we found that for some items the scoring range is not fully exhausted supporting the possible benefit of modifications. But the distributions for motor and phonic tic items often differ, which is a central problem for meaningful modifications of the anchor point descriptions since they refer to the assessment of both kinds of tics. Furthermore, our results suggest the benefits of an additional decrease of complexity by e.g., simplifying the anchor point descriptions or reducing the number of domains.

Moreover, alternative approaches such as regression models or models of probabilistic test theory might be useful to explore alternative structures for the YGTSS in the future. To further improve, standardize, and simplify the assessment of tics, utilization of alternative measurement modalities might additionally be helpful such as video records, tracking bracelets, and smartphone apps.

The following limitations of the study should be noted: (i) the sample consisted of children and adolescents up to the age of 16 only. Since no differences in the clinical characteristics of tics between children/adolescents and adults have been shown so far (8), we do not believe that this has influenced our results; (ii) although diagnosed with TS, a quarter of participants had no phonic tics during the week before the assessment. Nevertheless we decided to investigate the theoretically assumed two factor structure of the YGTSS with all participants, since the target group of the YGTSS includes all tic disorders (4). In line with this, sub-analyses including only the TS subsample resulted in similar findings; (iii) the mean overall impairment rating was relatively low suggesting that our sample comprised less affected children/adolescents; (iv) the available dataset did not allow to determine inter-rater reliability. Nevertheless, we assume a high quality of the raters since only leading European experts participated in this study and a rater training was performed (28); (v) EMTICS was designed as a multi-center study. Accordingly, merging of data may have caused errors or distortions despite standardization data management and cleaning. We have decided not to correct any site differences statistically (e.g., by using a regression model), since the present study focused on the quality of the YGTSS, which relies, among other things, on providing the same results under all conditions. Removing site differences would run the risk of artificially glossing over the results. Moreover, it cannot be ruled out that participants who were actually affected to a different extent were included in the different centers; (vi) due to the study design, translations of the YGTSS in various languages had to be used, although only for the Spanish version has been validated so far (52). Since large sample sizes are essential for accurate estimates (especially within the CFA and for Ω), separate analyses for each language were not possible. Thus, validations of all versions of the YGTSS are highly appreciated in further studies.

In conclusion, our findings suggest acceptable psychometric qualities of the YGTSS. The results showed good discriminant validity, confirming the strength of the YGTSS in differentiating between tic severity and comorbid conditions. Given the fact that the YGTSS is the current gold standard for tic assessment (5) and widely used in all kind of research including treatment studies, it is also important to be aware of its limitations. Our data revealed correlated measurement errors presumably due to unexplained intercorrelations between domains and anchor points. Although our results support at least some of the modifications already suggested by the recently published YGTSS-R (8), based on our data, additional investigations and modifications are desirable to further improve the quality of the scale. Furthermore, our results corroborate clinical practice of separate use of the TSS and the impairment rating instead of the sum score GSS.

## Data Availability Statement

The datasets presented in this article are not readily available because: prior consultation with the study coordinators of EMTICS is required. The raw data can be made available for the purpose of verifying the outcomes of the present study. Requests to access the datasets should be directed to Dr. Andrea Dietrich, a.dietrich@accare.nl.

## Ethics Statement

The studies involving human participants were reviewed and approved by the institutional review boards at all participating clinical centers in accordance with the ethical standards laid down in the 1964 Declaration of Helsinki and its later amendments. Written informed consent to participate in this study was provided by the participants' legal guardian/next of kin.

## Author Contributions

MH, KM-V, EJ, and BJ contributed to the conception and design of the study. AD, PH, CF, KM-V, and EJ contributed to the acquisition of data and organized the database. MH wrote the first draft of the manuscript. MH, KM-V, EJ, BJ, CF, AD, and PH contributed to the analysis and interpretation of data. All authors contributed to manuscript revision, read and approved the submitted version.

## Emtics Collaborative Group

EMTICS group members are Alan Apter^1^, Valentina Baglioni^2^, Juliane Ball^3^, Noa Benaroya-Milshtein^1^, Benjamin Bodmer^4^, Emese Bognar^5, 6^, Bianka Burger^7, 8^, Judith Buse^4^, Francesco Cardona^2^, Marta Correa Vela^9^, Nanette M. Debes^10^, Andrea Dietrich^11^, Maria Cristina Ferro^12^, Carolin Fremer^13^, Blanca Garcia-Delgar^14^, Mariangela Gulisano^15^, Annelieke Hagen^15, 16^, Julie Hagstrøm^17^, Tammy J. Hedderly^18^, Isobel Heyman^19^, Pieter J. Hoekstra^11^, Chaim Huyser^15, 16^, Marcos Madruga-Garrido^20^, Anna Marotta^21^, Davide Martino^22^, Pablo Mir^9^, Astrid Morer^14, 23, 24, 25^, Norbert Müller^7, 8^, Kirsten R. Müller-Vahl^13^, Alexander Münchau^26^, Peter Nagy^5, 27^, Valeria Neri^2^, Thaïra J.C. Openneer^11^, Alessandra Pellico^12^, Ángela Periañez Vasco^9^, Kerstin J. Plessen^17, 28^, Cesare Porcelli^21^, Marina Redondo^14^, Renata Rizzo^12^, Veit Roessner^4^, Daphna Ruhrman^1^, Jaana M.L. Schnell^7^, Anette Schrag^29^, Paola Rosaria Silvestri^2^, Liselotte Skov^10^, Tamar Steinberg^1^, Friederike Tagwerker Gloor^3^, Zsanett Tarnok^5^, Jennifer Tübing^30^, Victoria L. Turner^18^, Susanne Walitza^3^, and Elif Weidinger^7^.

^1^Child and Adolescent Psychiatry Department, Schneider Children's Medical Center of Israel, affiliated to Sackler Faculty of Medicine, Tel Aviv University, Petah-Tikva, Israel.

^2^University La Sapienza of Rome, Department of Human Neurosciences, Rome, Italy.

^3^Clinic of Child and Adolescent Psychiatry and Psychotherapy, University of Zurich, Zurich, Switzerland.

^4^Department of Child and Adolescent Psychiatry, Faculty of Medicine of the TU Dresden, Dresden, Germany.

^5^Vadaskert Child and Adolescent Psychiatric Hospital, Budapest, Hungary.

^6^Semmelweis University, 1st Department of Paediatrics, Budapest, Hungary.

^7^Department of Psychiatry and Psychotherapy, University Hospital, LMU Munich, Munich, Germany.

^8^Marion von Tessin Memory-Zentrum gGmbH, Munich, Germany.

^9^Unidad de Trastornos del Movimiento, Servicio de Neurología y Neurofisiología Clinica. Instituto de Biomedicina de Sevilla (IBiS), Hospital Universitario Virgen del Rocio/CSIC/Universidad de Sevilla, Seville, Spain.

^10^Paediatric Department, Herlev University Hospital, Herlev, Denmark.

^11^University of Groningen, University Medical Center Groningen, Department of Child and Adolescent Psychiatry, Groningen, The Netherlands.

^12^Child Neuropsychiatry Section, Department of Clinical and Experimental Medicine, School of Medicine, Catania University, Catania, Italy.

^13^Clinic of Psychiatry, Socialpsychiatry and Psychotherapy, Hannover Medical School, Hannover, Germany.

^14^Department of Child and Adolescent Psychiatry and Psychology, Institute of Neurosciences, Hospital Clinic Universitari, Barcelona, Spain.

^15^De Bascule, Academic Center for Child and Adolescent Psychiatry, Amsterdam, The Netherlands.

^16^Academic Medical Center, Department of Child and Adolescent Psychiatry, Amsterdam, The Netherlands.

^17^Child and Adolescent Mental Health Center, Mental Health Services, Capital Region of Denmark and University of Copenhagen, Copenhagen, Denmark.

^18^Evelina London Children's Hospital GSTT, Kings Health Partners AHSC, London, UK.

^19^Great Ormond Street Hospital for Children, and UCL Institute of Child Health, London, UK.

^20^Sección de Neuropediatría, Instituto de Biomedicina de Sevilla (IBiS), Hospital Universitario Virgen del Rocío/CSIC/Universidad de Sevilla, Seville, Spain.

^21^Azienda Sanitaria Locale di Bari, Mental Health Department, Child and Adolescent Service of Bari Metropolitan Area, Bari, Italy.

^22^Department of Clinical Neurosciences, University of Calgary, Calgary, Canada.

^23^Institut d'Investigacions Biomediques August Pi i Sunyer (IDIBAPS), Barcelona, Spain.

^24^Centro de Investigacion en Red de Salud Mental (CIBERSAM), Instituto Carlos III, Spain.

^25^University of Barcelona, Barcelona, Spain.

^26^Institute of Systems Motor Science, University of Lübeck, Lübeck, Germany.

^27^Bethesda Children's Hospital, Budapest, Hungary.

^28^Division of Child and Adolescent Psychiatry, Department of Psychiatry, University Medical Center, University of Lausanne, Lausanne, Switzerland.

^29^Department of Clinical Neurosciene, UCL Institute of Neurology, University College London, London, UK.

^30^Department of Neurology, University of Lübeck, Lübeck, Germany.

## Conflict of Interest

TJH and VT have received funding from the Guys and St Thomas' NHS Foundation Trust. IH has received research funding or support from the National Institute for Health Research Biomedical Research Centre at Great Ormond Street Hospital for Children NHS Foundation Trust and University College London. PM has received grants from the Instituto de Salud Carlos III (PI10/01674, PI13/01461), the Consejería de Economía, Innovación, Ciencia y Empresa de la Junta de Andalucía (CVI-02526, CTS-7685), the Consejería de Salud y Bienestar Social de la Junta de Andalucía. KM-V has received financial or material research support from the EU (FP7-PEOPLE-2012-ITN No. 316978), the German Research Foundation (DFG: GZ MU 1527/3-1), the German Ministry of Education and Research (BMBF: 01KG1421), the National Institute of Mental Health (NIMH), the Tourette Gesellschaft Deutschland e.V., the Else-Kröner-Fresenius-Stiftung, and Abide Therapeutics, Almirall Hermal GmbH, GW pharmaceuticals, Lundbeck, Syneos Health, and Therapix Biosciences Ltd. She has received consultant's honoraria from Abide Therapeutics, Allmiral, Boehringer Ingelheim International GmbH, Bionorica Ethics GmbH, CannaMedical Pharma GmbH, Canopy Grouth, Columbia Care, CTC Communications Corp., Eurox Deutschland GmbH, Global Praxis Group Limited, Lundbeck, Resalo Vertrieb GmbH, Sanity Group, STADAPHARM GmbH, Synendos Therapeutics AG, and Tilray. She was a consultant or advisory board member for Abide Therapeutics, The Academy of Medical Cannabis Limited, Alirio, Aphria Deutschland GmbH, CannaMedical Pharma GmbH, Bionorica Ethics GmbH, CannaXan GmbH, Canopy Growth, Columbia Care, CTC Communications Corp., Leafly Deutschland GmbH, Lundbeck, Nuvelution TS Pharma Inc., Resalo Vertrieb GmbH, Sanity Group, Syqe Medical Ltd., Therapix Biosciences Ltd., Tilray, and Wayland Group. She has received speaker's fees from Aphria Deutschland GmbH, Cogitando GmbH, Emalex, Eurox Deutschland GmbH, Ever pharma GmbH, PR Berater, Spectrum Therapeutics GmbH, Tilray, and Wayland Group. She has received royalties from Medizinisch Wissenschaftliche Verlagsgesellschaft Berlin, Elsevier, and Kohlhammer. She served as a Guest editor for Frontiers in Neurology on the research topic “The neurobiology and genetics of Gilles de la Tourette syndrome: new avenues through large-scale collaborative projects,” and is Associate editor for “Cannabis and Cannabinoid Research,” Editorial Board Member for “Medical Cannabis and Cannabinoids” and “MDPI-Reports,” and scientific board member for “Zeitschrift für Allgemeinmedizin.” AM has received research funding or support from the Possehl-Stiftung (Lübeck, Germany), the Margot und Jürgen Wessel Stiftung (Lübeck, Germany), the Tourette Syndrome Association (Germany), Interessenverband Tourette Syndrom (Germany), CHDI, Damp-Stiftung; Deutsche Forschungsgemeinschaft (DFG): projects 1692/3-1, 4-1, SFB 936, and FOR 2698 (project numbers 396914663, 396577296, 396474989), Innovationsausschuss of the Gemeinsamer Bundesausschuss: Translate NAMSE (structural support for the Lübeck Center for Rare Diseases); European Reference Network—Rare Neurological Diseases (ERN—RND). SW has received in the last 5 years royalties from Thieme Hogrefe, Kohlhammer, Springer, Beltz. Her work was supported in the last 5 years by the Swiss National Science Foundation (SNF), diff. EU FP7s, HSM Hochspezialisierte Medizin of the Kanton Zurich, Switzerland, Bfarm Germany, ZInEP, Hartmann Müller Stiftung, Olga Mayenfisch, Gertrud Thalmann, Vontobel, Unicentia, Erika Schwarz Fonds. Outside professional activities and interests are declared under the link of the University of Zurich www.uzh.ch/prof/ssl-dir/interessenbindungen/client/web/. AS has received research funding or support from University College London, National Institute of Health (NIHR), National Institute for Health Research ULCH Biomedical Research Centre, the International Parkinson and Movement Disorder Society (IPMDS), the European Commission, Parkinson's UK, GE Healthcare and the Economic and Social Research Council. Honoraria for consultancy from Biogen, Abbvie, Roche, Bial, GE Healthcare; and license fee payments from the University College London for the MSA-QoL, PSP-QoL, and PQolCarers. Royalties from Oxford University Press. The remaining authors declare that the research was conducted in the absence of any commercial or financial relationships that could be construed as a potential conflict of interest.
