# Supplementary material for: Yale Global Tic Severity Scale (YGTSS): Psychometric Quality of the Gold Standard for Tic Assessment Based on the Large-Scale EMTICS Study
Source: Front Psychiatry. 2021 Feb 25;12:626459. doi: 10.3389/fpsyt.2021.626459 (PMC7949908; doi:10.3389/fpsyt.2021.626459)
Supplement: Supplementary file 1 [file Data_Sheet_1.PDF]

Supplementary Material

Supplementary Table 1 Misspecification regarding uncorrelated errors

	MI	EPC	NCP	Power	Decision
Motor: Complexity ~ Phonic: Complexity	18.628	0.183	5.533	0.653	m**
Motor: Interference ~ Phonic: Interference	18.526	0.224	3.698	0.485	m**
Phonic: Frequency ~ Phonic: Intensity	16.015	0.185	4.657	0.579	m**
Motor: Interference ~ Overall Impairment	12.084	0.175	3.966	0.513	m**
Phonic: Frequency ~ Phonic: Complexity	11.032	-0.151	4.831	0.594	m**
Motor: Frequency ~ Motor: Complexity	6.916	-0.127	4.309	0.546	m**
Motor: Number ~ Motor: Complexity	6.793	0.125	4.361	0.551	m**
Motor: Interference ~ Phonic: Frequency	5.740	-0.094	6.458	0.719	m**
Motor: Complexity ~ Overall Impairment	5.239	-0.110	4.299	0.545	m**
Overall Impairment ~ Phonic: Interference	4.894	0.116	3.607	0.476	m**
Motor: Frequency ~ Motor: Intensity	4.187	0.127	2.597	0.364	m**
Motor: Number ~ Overall Impairment	4.136	-0.099	4.236	0.539	m**
Motor: Number ~ Phonic: Intensity	3.992	-0.081	6.095	0.695	m**
Motor: Frequency ~ Phonic: Frequency	3.566	0.077	5.941	0.683	i
Motor: Number ~ Motor: Frequency	3.453	0.103	3.258	0.439	i

Abbreviations: MI=modification indice; EPC=expected parameter change; NCP=noncentrality parameter; m=model misspecification; i=Inconclusive on model misspecification; results for decision “no model misspecification” not shown; **p< .001, For detailed description of the analysis see Saris et al. (35).
